# Comparing the oncologic outcomes of local tumor destruction vs. local tumor excision vs. partial nephrectomy in T1a solid renal masses: a population-based cohort study from the SEER database – correspondence

**DOI:** 10.1097/JS9.0000000000001894

**Published:** 2024-07-02

**Authors:** Qunlong Liu, Ming Chen, Jianping Wu, Weipu Mao

**Affiliations:** a Department of Obstetrics and Gynecology, The People’s Hospital of Yingshang, Anhui China; b Department of Urology, Zhongda Hospital, Southeast University, Nanjing, People’s Republic of China

*Dear Editor*,

We are interested in the article titled ‘Comparing the oncologic outcomes of local tumor destruction vs. local tumor excision vs. partial nephrectomy in T1a solid renal masses’ written by Guo *et al*.^1^. The authors compared three different treatment modalities, local tumor destruction (LTD), local tumor excision (LTE), and partial nephrectomy (PN), analyzing cancer-specific survival (CSS) and overall survival (OS) of patients using data from the 2000–2019 Surveillance, Epidemiology, and End Results (SEER) database for T1a solid renal masses. Their findings suggest that LTE is the most effective surgical modality, followed by PN and LTD. However, we have some concerns regarding the study.

In Figure 1 and Supplementary Figures S1 and S2, the authors presented the OS and CSS curves post-propensity score matching (PSM) for LTD vs. LTE, LTD vs. PN, and LTE vs. PN, respectively. The 5-year OS rates for the LTD, LTE, and PN groups were 0.819, 0.873, and 0.846, respectively, and the 10-year OS rates were 0.306, 0.419, and 0.321, respectively. The 5-year CSS rates were 0.661, 0.731, and 0.773, respectively, and the 10-year CSS rates were 0.181, 0.262, and 0.250, respectively. Additionally, we noted that all patients died within 200 months of follow-up (cumulative survival=0), which contradicts clinical expectations, leading us to suspect that ‘survival’ and ‘death’ may have been inverted in the survival curves.

**Figure 1:**
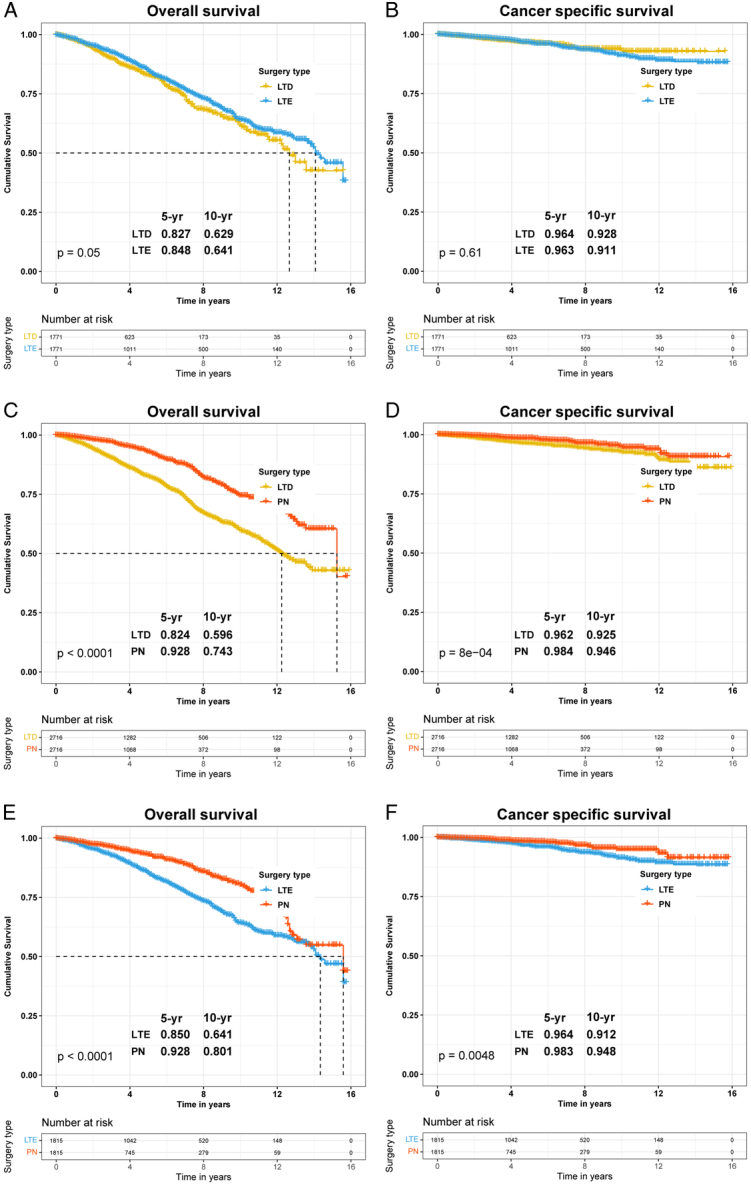
Overall survival (OS) and cancer-specific survival (CSS) curves between the local tumor destruction (LTD) group, local tumor excision (LTE) group, and partial nephrectomy (PN) group after 1:1 propensity score matching (PSM) in patients with T1a renal cell carcinoma. (A) OS in LTD vs. LTE post-PSM; (B) CSS in LTD vs. LTE post-PSM; (C) OS in LTD vs. PN post-PSM; (D) CSS in LTD vs. PN post-PSM; (E) OS in LTD vs. PN post-PSM; (F) CSS in LTD vs. PN post-PSM.

We reviewed recent studies on patients with T1a renal cell carcinoma (RCC) from PubMed. Wang *et al*.^2^ analyzed clinical data of 27 502 patients with T1aN0M0 RCC from the SEER database (2004–2015), revealing that the 10-year OS exceeds 70% and the 10-year CSS exceeds 90%. Additionally, Sorce *et al*.^3^ utilizing patients with T1a RCC from the SEER database, found that LTD leads to higher cancer-specific mortality (CSM) compared to PN, with a 10-year CSM of 8.7% for LTD and 5.5% for PN. Comparison of different resection techniques during PN, such as resection, enucleoresection or enucleation, might impact prognosis more meaningful^4^.

We extracted clinical data for 33 516 patients with T1a RCC from the SEER database (2004–2019) based on the authors’ inclusion and exclusion criteria. Among these, 2749, 1820, and 28 946 received LTD, LTE, and PN, respectively. Kaplan–Meier survival curves for the three groups (LTD, LTE, and PN) (Supplementary Fig. S1, Supplemental Digital Content 1, http://links.lww.com/JS9/C956) showed 5-year OS rates of 0.819, 0.850, and 0.942, respectively, and 10-year OS rates of 0.589, 0.637, and 0.840, respectively (Supplementary Fig. S1A, Supplemental Digital Content 1, http://links.lww.com/JS9/C956). The 5-year CSS rates were 0.962, 0.964, and 0.989, respectively, and the 10-year CSS rates were 0.924, 0.912, and 0.970, respectively (Supplementary Fig. S1B, Supplemental Digital Content 1, http://links.lww.com/JS9/C956). Additionally, statistical differences in OS were found between the LTD and LTE groups (*P*=0.00061, Supplementary Fig. S2A, Supplemental Digital Content 2, http://links.lww.com/JS9/C957), but not in CSS (*P*=1.000, Supplementary Fig. S2B, Supplemental Digital Content 2, http://links.lww.com/JS9/C957). Differences in OS and CSS were statistically significant between LTD and PN, as well as between LTE and PN groups (LTD vs. PN: OS, *P*<0.0001, Supplementary Fig. S2C, Supplemental Digital Content 2, http://links.lww.com/JS9/C957; CSS, *P*<0.0001, Supplementary Fig. S2D, Supplemental Digital Content 2, http://links.lww.com/JS9/C957; LTE vs. PN: OS, *P*<0.0001, Supplementary Fig. S2E, Supplemental Digital Content 2, http://links.lww.com/JS9/C957; CSS, *P*<0.0001, Supplementary Fig. S2F, Supplemental Digital Content 2, http://links.lww.com/JS9/C957). Furthermore, multivariate Cox regression indicated an 18.9% decrease in OS risk for LTE compared to LTD, with no difference in CSS risk, while PN had a 52.5% lower OS risk and 48.9% lower CSS risk. Compared to LTE, PN showed a 38.8% lower OS risk and a 48.6% lower CSS risk (Supplementary Table S1, Supplemental Digital Content 3, http://links.lww.com/JS9/C958).

Subsequently, we conducted PSM analysis for the three groups (Supplementary Materials and Methods, Supplemental Digital Content 4, http://links.lww.com/JS9/C959) and plotted the corresponding Kaplan–Meier survival curves. The analyses revealed no statistical difference in OS and CSS between LTD and LTE (OS, *P*=0.050, Fig. 1A; CSS, *P*=0.610, Fig. 1B). Significant differences in both OS and CSS were observed between LTD and PN, and LTE and PN (LTD vs. PN: OS, *P*<0.0001, Fig. 1C; CSS, *P*=0.0008, Fig. 1D; LTE vs. PN: OS, *P*<0.0001, Fig. 1E; CSS, *P*=0.0048, Fig. 1F). Furthermore, multivariate Cox regression analysis showed a 25.1% decrease in OS risk for LTE compared to LTD, with no statistical difference in CSS risk, while PN had a 49.2% lower OS risk and a 37.7% lower CSS risk. Compared to LTE, PN showed a 33.6% lower OS risk and a 35.3% lower CSS risk (Supplementary Table S1, Supplemental Digital Content 3, http://links.lww.com/JS9/C958).

In conclusion, for patients with clinical T1a RCC, PN is the optimal surgical approach compared to LTD and LTE. Although LTD and LTE are at a disadvantage in terms of OS and CSS, they may be effective options for patients where reducing complications and preserving kidney function are more important. However, when LTD and LTE are considered as treatment options, thorough communication with the patient is necessary.

## Ethical approval

Not applicable.

## Consent

Not applicable.

## Source of funding

This work was supported by the Natural Science Foundation of Jiangsu Province (BK20230842) and the Research Personnel Cultivation Programme of Zhongda Hospital Southeast University (CZXM-GSPRC60).

## Author contribution

Q.L. and W.M.: data collection and writing the paper; M.C. and J.W.: study concept and review and editing.

## Conflicts of interest disclosure

There are no conflicts of interest.

## Research registration unique identifying number (UIN)

Not applicable.

## Guarantor

Weipu Mao.

## Data availability statement

The datasets used and/or analyzed during the current study are available from the corresponding author on reasonable request.

## Provenance and peer review

Not applicable.

## Supplementary Material

SUPPLEMENTARY MATERIAL
