# Supplementary figures and images for: Comparing the oncologic outcomes of local tumor destruction vs. local tumor excision vs. partial nephrectomy in T1a solid renal masses: a population-based cohort study from the SEER database – correspondence
Source: Int J Surg. 2024 Jul 2;110(10):6833–5. doi: 10.1097/JS9.0000000000001894 (PMC11486955; doi:10.1097/JS9.0000000000001894)

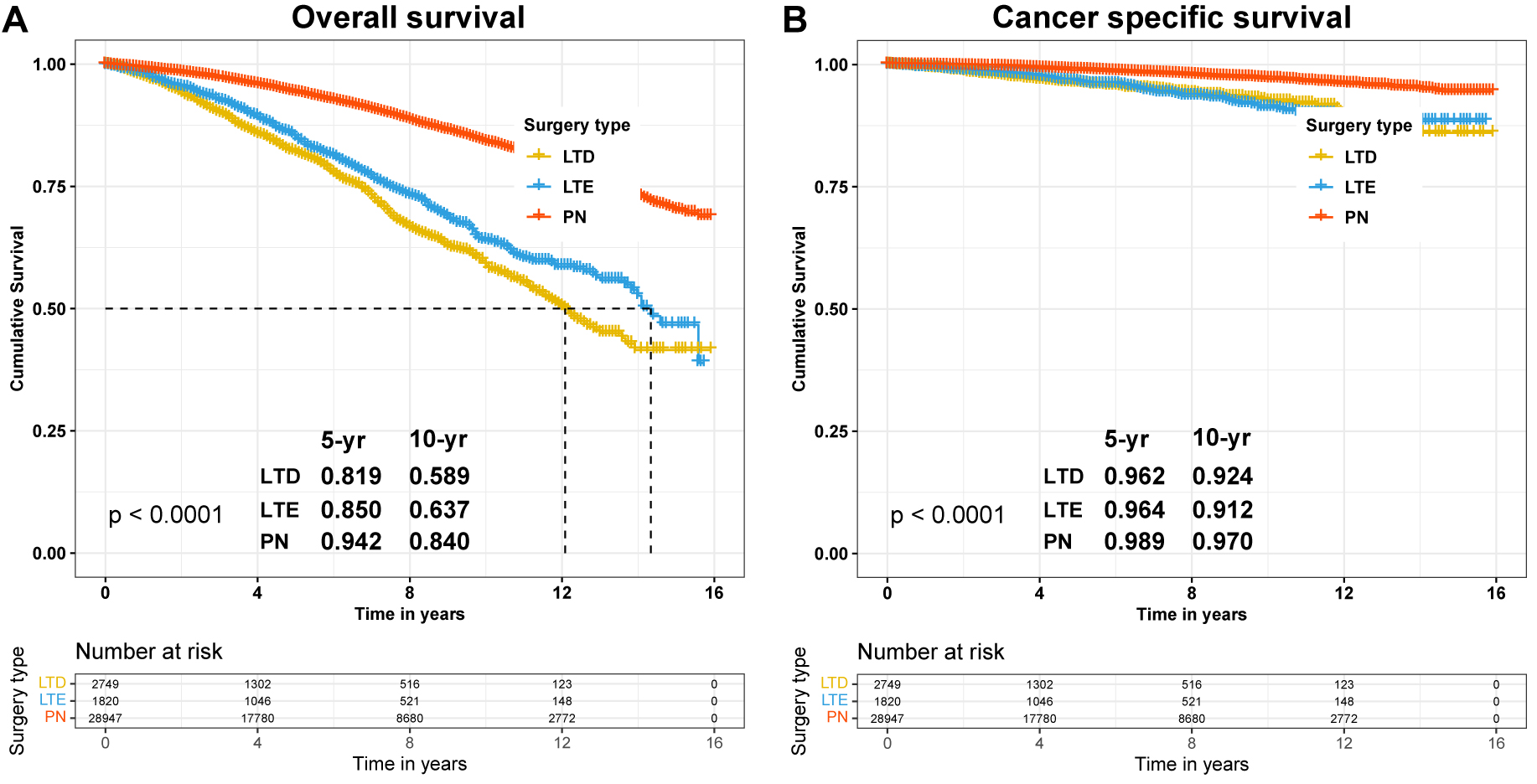

Supplement: SUPPLEMENTARY MATERIAL [file js9-110-6833-s001.jpg]

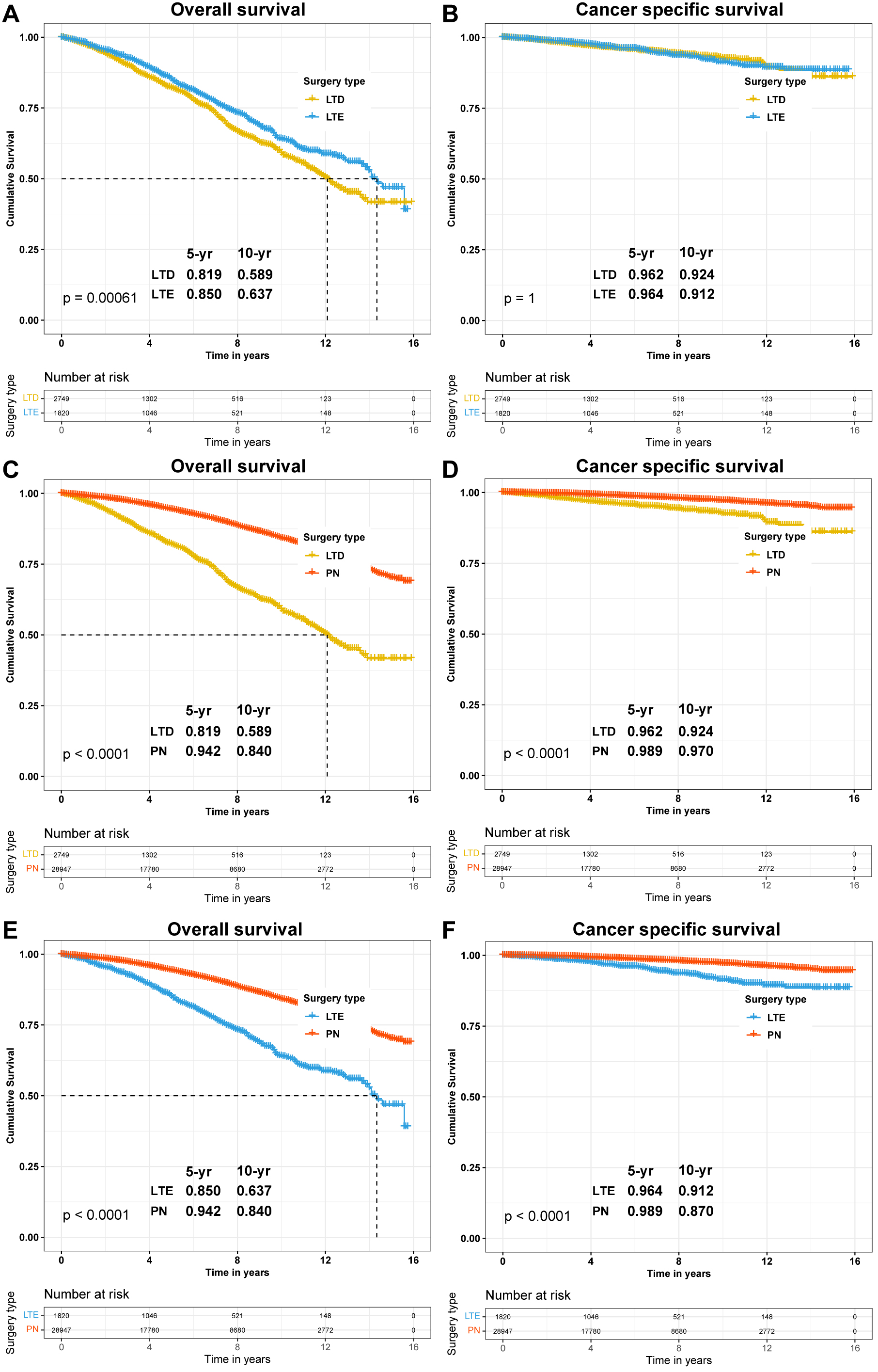

Supplement: SUPPLEMENTARY MATERIAL [file js9-110-6833-s002.jpg]
