# Supplementary material for: Comparing the oncologic outcomes of local tumor destruction vs. local tumor excision vs. partial nephrectomy in T1a solid renal masses: a population-based cohort study from the SEER database – correspondence
Source: Int J Surg. 2024 Jul 2;110(10):6833–5. doi: 10.1097/JS9.0000000000001894 (PMC11486955; doi:10.1097/JS9.0000000000001894)
Supplement: SUPPLEMENTARY MATERIAL [file js9-110-6833-s003.docx]

**Table S1. Multivariate analyses of factors associated with overall survival (OS) and cancer-specific survival (CSS) before and after 1:1 propensity score matching.**

| **Characteristics** | **Before propensity score matching** | | **After propensity score matching** | |
| --- | --- | --- | --- | --- |
|  | **Hazard Ratio (95% CI)** | ***P* value** | **Hazard Ratio (95% CI)** | ***P* value** |
| LTD vs LTE |  |  |  |  |
| Overall survival |  |  |  |  |
| LTD | Reference |  | Reference |  |
| LTE | 0.811 (0.712-0.924) | 0.002 | 0.749 (0.634-0.885) | 0.001 |
| Cancer-specific survival |  |  |  |  |
| LTD | Reference |  | Reference |  |
| LTE | 1.020 (0.759-1.370) | 0.896 | 0.816 (0.565-1.180) | 0.281 |
| LTD vs PN |  |  |  |  |
| Overall survival |  |  |  |  |
| LTD | Reference |  | Reference |  |
| PN | 0.475 (0.430-0.524) | <0.001 | 0.508 (0.421-0.613) | <0.001 |
| Cancer-specific survival |  |  |  |  |
| LTD | Reference |  | Reference |  |
| PN | 0.511 (0.403-0.649) | <0.001 | 0.623 (0.413-0.939) | 0.024 |
| LTE vs PN |  |  |  |  |
| Overall survival |  |  |  |  |
| LTE | Reference |  | Reference |  |
| PN | 0.612 (0.547-0.684) | <0.001 | 0.664 (0.522-0.845) | 0.001 |
| Cancer-specific survival |  |  |  |  |
| LTE | Reference |  | Reference |  |
| PN | 0.514 (0.401-0.659) | <0.001 | 0.647 (0.392-1.070) | 0.090 |

**Abbreviations:**

OS, Overall survival; CSS, Cancer-specific survival; CI, confidence interval; LTD: local tumor destruction; LTE: local tumor excision; PN: partial nephrectomy.
