# Supplementary material for: Comparing the oncologic outcomes of local tumor destruction vs. local tumor excision vs. partial nephrectomy in T1a solid renal masses: a population-based cohort study from the SEER database – correspondence
Source: Int J Surg. 2024 Jul 2;110(10):6833–5. doi: 10.1097/JS9.0000000000001894 (PMC11486955; doi:10.1097/JS9.0000000000001894)
Supplement: SUPPLEMENTARY MATERIAL [file js9-110-6833-s004.docx]

**Comparing the oncologic outcomes of local tumor destruction vs. local tumor excision vs. partial nephrectomy in T1a solid renal masses: a population-based cohort study from the SEER database –** **correspondence**

**Material and methods**

Study population

We obtained clinical data on 33,516 patients diagnosed with T1a renal cell carcinoma (RCC) between 2004 and 2019 using SEER*Stat 8.4.3 software, based on the authors' inclusion and exclusion criteria from SEER Research Data, 17 Registries, Nov 2021 Sub (2000-2019).

Study variables and definitions

We included variables such as gender, age (18-39, 40-59, 60-79 and 80+), race (White, Black, Asian or Pacific Islander, American Indian/Alaska Native and Unknown), laterality (Left and Right), grade (Grade I, Grade II, Grade III, Grade IV and Unknown), histology (ccRCC, pRCC, chRCC and nosRCC) and tumor size. OS was defined using the ‘Vital status recode’ variable, classifying ‘Alive’ individuals as survivors and ‘Dead’ individuals as deceased. CSS was defined using ‘SEER cause-specific death classification’, where ‘Dead (attributable to this cancer dx)’ indicated cancer-specific death, and others were considered survivors.

Statistical analysis

Continuous data were expressed as mean ± standard deviation (SD) and categorical data as number (%). T-tests were used for continuous variables and chi-square tests for categorical variables. Multivariate Cox regression assessed the relationship between different surgical approaches and OS and CSS, calculating adjusted hazard ratios (aHRs) and 95% confidence intervals (CIs). Additionally, a 1:1 PSM analysis was conducted by "MatchIt" R package to balance differences among the LTD, LTE and PN groups, adjusting for gender, age, race, laterality, grade, histology and tumor size. To further validate the results, a reanalysis was performed on the PSM-adjusted data. All data and figures were processed and analysed using R software (version 3.5.3) and SPSS software (version 25.0). Statistical significance was set at p < 0.05.
